# Cell-Block cytology in diagnosis of primary central nervous system lymphoma: A case report

**DOI:** 10.1097/MD.0000000000019598

**Published:** 2020-04-03

**Authors:** Kaiyuan Huang, Lei Zhou, Ying Tong

**Affiliations:** The First Affiliated Hospital, College of Medicine, Zhejiang University, Hangzhou, Zhejiang Province, China.

**Keywords:** Cell-block, cell-free DNA, cerebrospinal fluid, liquid biopsy, Primary Central Nervous System Lymphoma

## Abstract

**Introduction:**

Primary Central Nervous System Lymphoma (PCNSL) remains a diagnostic challenge due to the variable clinical manifestations. Liquid biopsies, particularly those involving cell-free DNA (cfDNA) from plasma, are rapidly emerging as important and minimally invasive adjuncts to traditional biopsies. However, conventional pathology may be still essential to obtain a diagnosis.

**Patient concerns:**

A 56-year-old woman presented with a progressive headache, dizziness, blurred vision, and lower limbs weakness with dysesthesia. Atypical clinical and radiological presentations, previous empirical treatment in another hospital, together with the patient's refusal to stereotactic brain biopsy made it challenging to diagnose. Her status deteriorated continuously during hospitalization.

**Diagnosis:**

Lumber punctual was performed, and CSF cytological analysis revealed malignancy cells with a high nuclear-cytoplasmic ratio. However, these cells were too loose to perform immunohistochemical stains. Genetic aberrations detections with CSF and peripheral blood sample were also inconclusive. We made a “cell-block” using the sedimentary cells collected from CSF collected through multiple aspirations via an Omaya reservoir. We further performed cytopathological and immunohistochemical analysis using this “cell-block,” which finally confirmed the diagnosis of diffuse large-B cell PCNSL.

**Interventions:**

Intracranial chemotherapy began afterwards (MTX 15 mg and dexamethasone 5 mg, twice per weeks).

**Outcomes:**

Unfortunately, this patient was dead 2 weeks later due to severe myelosuppression and secondary septic shock.

**Conclusion:**

We provided “cell-block” method, which collects cell components from large amount of CSF for cytology and immunohistochemical analysis. “Cell-block” cytology can be an alternative diagnostic method in diagnosis of PCNSL.

## 1 Introduction

Primary Central Nervous System Lymphoma (PCNSL) is a rare extranodal non-Hodgkin lymphoma (NHL), which can involve the brain, eye leptomeninges, and rarely spinal cord without evidence of systemic disease.^[1]^ It remains a diagnostical challenge as PCNSL may present with diverse neurological symptoms.^[2–4]^ Radiologically, PCNSL lesions most typically enhance homogeneously on T1-weighted magnetic resonance imaging (MRI) and appear T2-hypointense, but high variability in MRI features is commonly encountered.^[2]^ Besides, these MRI findings can mimic high-grade gliomas, metastatic tumors, tumefactive demyelinating lesions, or infectious and granulomatous diseases.^[5,6]^ Some recent studies suggest a role of advanced MRI and metabolic imaging such as fluorodeoxyglucose positron emission tomography (FDG-PET) in distinguishing PCNSL from these entities.^[7,8]^ However, the histological analysis of biopsy material is still regarded as a gold standard. Unfortunately, major complications can arise during the procedure of a brain biopsy, such as intracranial hemorrhage or functional impairment.^[9]^

The NCCN guideline for PCNSL also suggests 15 to 20 mL spinal CSF sampling to increase diagnostic yield under a safe condition. CSF analysis should include flow cytometry and CSF cytology and possibly gene rearrangements. However, the predictive value is still very low due to the sparsity of malignant cells in the CSF. Presently, liquid biopsies often use cerebrospinal fluid (CSF) for cytomorphologic and flow cytometric analysis in PCNSL diagnosis.^[9]^ Liquid biopsy also is a real-time sampling of circulating tumor cells, nucleotides from biofluids, which offers a promising, less-invasive mean to obtain tumor genetics and monitor tumor evolution in patients with both primary and secondary CNS malignancies.^[2,9–11]^ Presently, mutations in specific genes (e.g., MYD88, PIM1, ATM, and TP53), dysregulation in signaling pathways (e.g., JAK/STAT, NF-kB, toll-like receptor, and B-cell receptor), and some translocations and copy number alterations (e.g., 9p.24/PD-L1/PD-2) has been identified as new biomarkers for PCNLS diagnosis, which further expand the diagnostic armamentarium.^[6,9,12,13]^ Emerging data suggest a role for CSF molecular analysis in improving the sensitivity and specificity of liquid biopsies. Some biomarker may even predict prognosis and response to treatments.^[3,9]^

However, it is incapable of concluding diagnose purely based on molecular diagnosis. Thus, a special CSF procedure may need to provide conventional pathological supports. Here, we present a case, in which a PCNSL patient with atypical clinical and radiological presentations failed to conclude a diagnosis by gene sequencing. The final diagnosis was achieved by a “cell-block” method and subsequent classic histopathological analysis.

## 2 Case presentation

**Figure 1:**
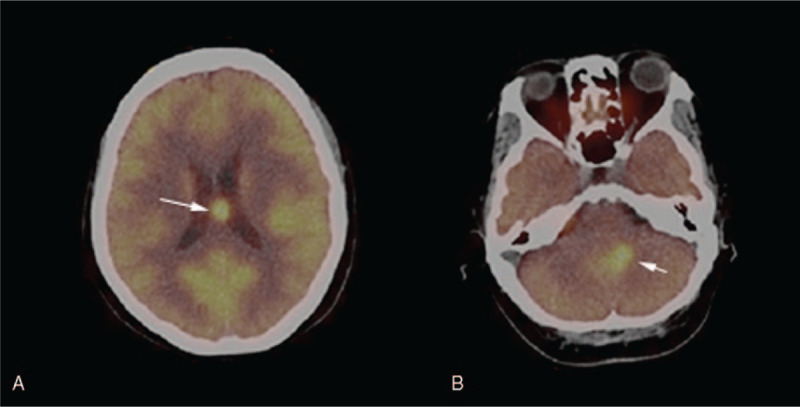
FDG-PET performed 4 months before this presentation showed that FDG uptake increased in the callosal area with a maximal SUV of 16.5 (panel A), and the area abutting the fourth ventricle with a maximal SUV of 16.2 (panel B).

A 56-year-old woman presented with a progressive headache, dizziness, blurred vision, and lower limbs weakness with dysesthesia. Her symptoms initiated four months before this presentation, and she went to the local hospital for examination. Gadolinium-enhanced contrast MRI revealed multiple intracranial mass with ring enhancement. Metastatic malignancy was the primary consideration. She underwent FDG-PET scan afterward, which showed an increased FDG uptake in the callosal area with a maximal standardized uptake value (SUV) of 16.5, and areas abutting the fourth ventricle with a maximal SUV of 16.2 (Fig. 1). However, PET-CT did not reveal any extracranial lesion. Spinal MRI revealed Chiari I malformation without lesions found at that time. Laboratory tests, including red-cell count, white-cell count, platelet count, renal- and liver function tests, were unrevealing except a decreased CD4+, CD8+, and CD3+ lymphocytes. The patient denied stereotactic biopsy, but she received radiotherapy for treatment of her lesions. Tumors margin was covered by 50% isodose line, and a total 2000 cGy in five fractions was delivered to each lesion (6 lesions in total). Her dizziness improved after treatment, while her blurred vision remained.

Two months before this presentation, her dizziness and gait imbalance recurred, in addition to a new onset headache and circumoral formication. Repeated gadolinium enhanced contrast MRI revealed diffuse lesion occupied areas around right cisterna ambiens, cerebellopontine angle areas, and the occipital lobe. She received another brain radiotherapy afterward. A total of 3600 cGy was given (200 cGy per fractions, 18 fractions in total), and she was in largely remission when discharged.

Two weeks before this presentation, she began to complain progressively intensifying low back pain, lower limb ache, and dysesthesia. Spinal MRI after administration of gadolinium showed multiple lesions occupied the whole spinal cord. The spinal spread of intracranial malignancy was considered. She was then referred to our department.

Her medical history was notable for coronary heart disease, second-degree atrioventricular block, hyperlipidemia, pulmonary tuberculosis, chronic HBV infection, and chronic atrophic gastritis. Medications on presentation were atorvastatin, gabapentin, caltrate, and lactulose.

She reported no fever, abdominal pain, chest pain, palpitations, or dyspnea.

On examination, her vital sign was normal, and she was alert, her vision was blurred, the hearing of her left ear was deceased, her muscle weakness of her lower and upper limbs was Grade II and Grade III, respectively. The muscular tension was normal, as well as the tendon reflexes. There was no neck stiffness and other sign of meningeal irritation.

**Figure 2:**
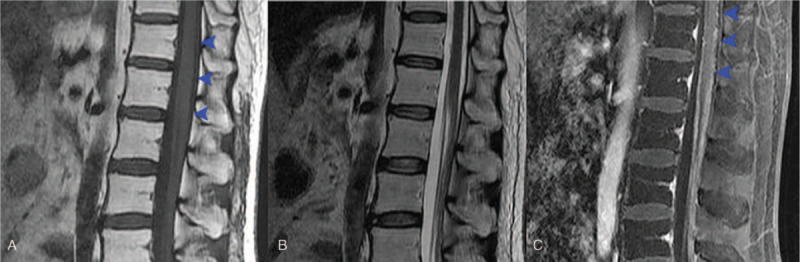
Spinal MRI revealed diffuse thickening of the dura matter and multiple tuberous lesion, which were enhanced after gadolinium administration (panel A: T1-weighted, panel B: T2-weighted, panel C: gadolinium contrast enhanced T1-weighted).

**Figure 3:**
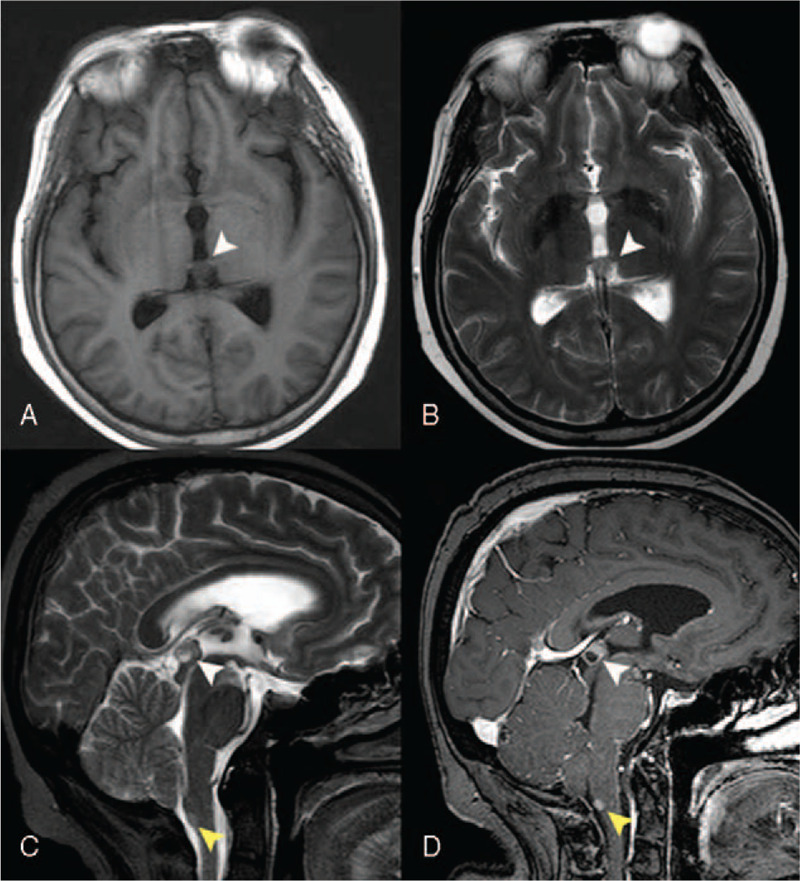
Cranial MRI showed an isointense nodular lesion in the pineal region on T1-weighted (panel A) and T2-weighted phases (panel B), which lead to mild hydrocephalus. Sagittal view of T2-weighted MRI also revealed a hyperintense lesion in the dorsal medulla (panel C, yellow arrowhead). Both two lesions were enhanced after gadolinium enhancement.

Lumbar puncture was performed, and laboratory examination of the cerebrospinal fluids (CSF) reveals decreased chloride (110 mmol/L, normal range 120–131 mmol/L) and glucose (0.0 mmol/L, normal range 2.5–4.5 mmol/L), and increased protein (6.870 g/L, normal rage 0.150–0.450 g/L), erythrocytosis (10/μL) and leukocytosis (160/μL, neutrophil 10%, lymphocyte 55%, and endothelial cell 35%). CSF smear revealed malignancy cells with a high nuclear-cytoplasmic ratio, whereas these cells were too loose for immunohistochemical stains. On hospitalization, her condition was deteriorating continuously. She began to show multiple cranial nerve palsy, including right ptosis and dysphagia. Repeated cranial and spinal MRI revealed malignancy progression (Figs. 2 and 3).

To determine the malignancy, CSF and peripheral blood sample were sent for further genetic sequencing and detection. The results from CSF showed genetic variation of ABL1, AR, CDK6, ERBB2, ERBB4, FGFR2, FGFR3, FLT3, HRAS, JAK2, KDR, MET, MPL, MYC, NTRK1, PDGF RB, PIK3C A, PTEN, RET, SMAD4, SMAR CB1, SMO, STK11, TP53, and VHL. The result from peripheral blood showed a genetic variation of MYC, PDGFRA, PIK3R1, and TP53. No gene rearrangement and copy number variation were detected. However, these results failed to certain the malignancy histogenesis. Therefore, alternative diagnostic method was wanted.

**Figure 4:**
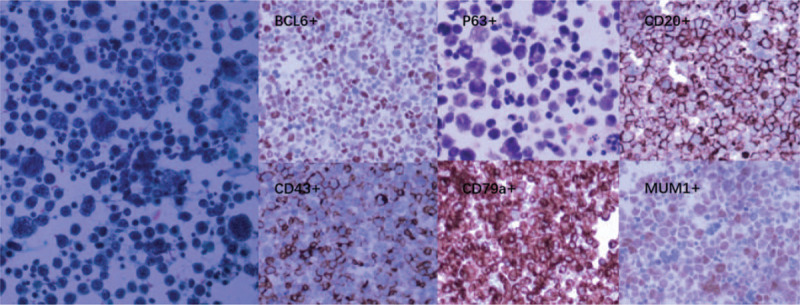
Microscopy of the “cell-block” revealed numerous malignant cells with a high nuclear-cytoplasmic ratio, which further confirmed the diagnosis of large B cell lymphoma by immunohistochemical stains (∗40∗10).

In the next days, this patient experienced increased intracranial pressure caused by hydrocephalus. Thus, we placed an Omaya reservoir for daily CSF drainage. We aspirated 10 to 15 mL CSF from the Omaya reservoir to decrease intracranial pressure. Considering the number of malignant cells in CSF sample obtained by each lumbar puncture was too few for immunohistochemical stain, we collected the daily drained CSF, and centrifuged the cell contents in the CSF. After we collected abundant CSF cells (about 0.2 mL), we made a compact cell-block processed with paraffin wax just like processing normal biopsied tissue specimen, and further immunohistochemical stains were performed (Fig. 4). The results showed CK(pan)(−), Ki-67(+++,90%), P63(partly+), ALK(−), Napsin A(−), CD30(−), CD34(−), CD43(+), MPO(−), CD2(−), CD3(−), CD4(−), CD7(−), CD5(−), CD20(+), CD79a(+), CD10(−), Bcl-6(+), MUM1(+), suggesting the diagnosis of diffuse large-B cell lymphoma (non-GCB type).

Afterward, this patient began her intracranial chemotherapy (MTX 15 mg and dexamethasone 5 mg, twice per weeks). Unfortunately, she was dead 2 weeks later due to severe myelosuppression and secondary septic shock during the process of chemotherapy.

## 3 Discussion

Primary Central Nervous System Lymphoma is a rare extranodal NHL, involving the brain, eye, leptomeningitis, and rarely spinal cord.^[3,14]^ The diagnosis of CNS lymphoma is always challenging. Presently, it is mainly based on clinical characteristics, imaging, CSF analysis, and brain biopsy.^[2,13,15–17]^ Magnetic resonance imaging is the cornerstone for PCNSL lesions determination, which most typically enhance homogeneously on T1-weighted (MRI) and appear T2-hypointense, but high variability in MRI features is commonly encountered.^[3]^Advanced MRI (MR diffusion, spectroscopy, and perfusion) and metabolic imaging, such as FDG-PET may help to distinguish PCNSL with other entities, such as high-grade gliomas, tumefactive demyelinating lesions, or infectious and granulomatous diseases.^[3,16]^Multiple MRIs and PET-CT were applied in this case. However, the radiological features were atypical regarding both the manifestation and location (multiple lesions, unusual places, and spinal involvement), which was more consistent with metastatic CNS malignancy than PCNSL.^[18,19]^

For PCNSL, histological analysis through a brain biopsy or liquid biopsy of biopsy material is still regarded as the gold standard.^[9]^ While brain biopsy provides histologic and genetic information, it is an invasive procedure with the risk of major complications. Brain biopsy is not conducive to obtaining multiple samples over time, and previous treatments may alter the diagnostic yield of biopsies, such as steroids and radiotherapies.^[2,10,20,21]^ Alternatively, the liquid biopsy is a promising, less-invasive means to obtain the histologic diagnosis, from multi-analysis of the cytology, flow cytometry, molecular markers, and specific gene mutations.^[2,13]^ Among these CSF analyses, cytology or cytopathology (leukocyte count and differentiation, cytomorphology, and immunohistochemistry) entails the gold standard.^[13]^ Unfortunately, this method has a low diagnostic yield as often no malignant cell detected, or cells are too lose or too lytic for proper analysis.^[9,13]^ Despite malignant cells were found in the initial CSF analysis in our case, the number was not abundant to perform the immunohistochemistry. As is often the case, diagnosis of PCNSL from CSF cytology occurs in only 10% to 20% patients, as malignant cells detected are too loosely or too lytic for proper analysis.^[2,22]^ Flow cytometry, a method of immunophenotyping (detection for clonal B-cell population), can increase the sensitivity in the diagnosis of PCNSL, which usually works together with cytology.^[23,24]^ In our case, flow cytometric analysis was not performed due to initial suspicion of metastatic malignancy. However, sensitivity is disappointing even for this analysis approach, and detection of clonal B-cell populations does not distinguish between lymphoma entities.^[9]^

The advent of lymphoma-specific mutations and unique combinations of biomarkers (cytokines, receptors, immunoglobulins, and miRNAs) that can be tested in CSF further increase the diagnostic value of this liquid biopsy for PCNSL.^[25,26]^ Multiple studies have tried to identify a molecular signature specific for PCNSL, using many different analysis strategies on tumor tissue. Interleukin (IL)-10 with its receptors, IL-6 and CXCL13 are shown to be upregulated in the CSF of patients with PCNSL, which are related to lymphoid cells growth and immunity regulation.^[25,27]^ Osteopontin and neopterin are proinflammatory cytokines involved in immune cell activation and B-cell migration and proliferation, which are significantly higher in patients with PCNSL than in those with other CNS disorders.^[12,28]^ microRNAs (miRNAs) are very promising biomarkers as well, not only for diagnostic purposes but also for monitoring therapy response.^[13]^ Furthermore, emerging data suggest a role for gene aberrations in predicting prognosis and response to treatments. Presently, genetic aberrations reported in PCNSL include CARD11, CD79B, CDKN2A (9p21; p16), ETV6, MYD88, PIM1, PRDM1, TBL1XR1, TNFAIP3(A20), and TOX.^[9,16]^ Half of the selected genes are involved in the NFκB pathway (CARD11, CD79B, MYD88, TBL1XR1, and TNFAIP3), while the other half is not (CDKN2A, ETV6, PIM1, PRDM1, and TOX).^[9]^ Unfortunately, none of these genetic aberrations were detected in this patient. The results of CNS specific gene mutation detection are often inconclusive and hard to interpret in clinical practice, on which the diagnostic criteria based still has a long way to validation.

The final diagnosis of our case relied on the cytopathologic and combined immunohistochemistry analysis of the “cell-block,” which made from CSF cells collected from repeatedly aspirated patient's CSF. To the best of our knowledge, this is the first case demonstrating such a “cell-block” methods. As the major constraint is removed (the hypocellular composition of CSF), classical histopathological diagnostic methods can function effectively in liquid biopsy, such as cytopathology and immunohistochemistry analysis.^[29]^ Therefore, this “cell-block” method can improve the effectiveness of liquid biopsy, and especially useful in cases where the molecular analysis is unavailable or inconclusive.

However, it is not known if this methods help if PCNSL did not invade the CSF circulation system. As this is only a case report, further clinical studies should performed to address the sensitivity and specificity of this cell-block method.

## 4 Conclusion

This case demonstrated a safe, easy-practicing and cost-effective method for CSF procedure for PCNSL diagnosis. Presently, molecular diagnosis is still far from an essential diagnostic method for PCNSL; this “cell-block” method is especially useful in the scenario where brain biopsy, molecular analysis are unavailable or inconclusive.

## Author contributions

**Data curation:** Lei Zhou.

**Writing – original draft:** Kaiyuan Huang.

**Writing – review & editing:** Ying Tong.
